# TMJ Replacement in Degenerative Disease: A Systematic Review

**DOI:** 10.3390/jcm14020580

**Published:** 2025-01-17

**Authors:** Víctor Ravelo, Erick Vargas, Henry García Guevara, Roberto Sacco, Pablo Navarro, Sergio Olate

**Affiliations:** 1 Grupo de Investigación de Pregrado en Odontología (GIPO), Facultad de Ciencias de la Salud, Universidad Autónoma de Chile, Temuco 4780000, Chile; victor.ravelo.s@gmail.com (V.R.);; 2 PhD Program and Center of Morphological and Surgical Research (CEMyQ), Universidad de La Frontera, Temuco 4811230, Chile; 3 Division of Oral and Maxillofacial Surgery, C.H.M Hospital, Chillán 3810525, Chile; evargas86@gmail.com; 4 Fellowship Program in Orthognathic and Complementary Facial Surgery, Universidad de La Frontera, Temuco 4811230, Chile; 5 Division for Oral and Maxillofacial Surgery, Hospital Ortopedico Infantil, Caracas 1060, Venezuela; henryagg@gmail.com; 6 Department of Oral Surgery, La Floresta Medical Institute, Caracas 1060, Venezuela; 7 Department of Oral Surgery, Faculty of Dentistry, Oral & Craniofacial Sciences, King’s College London, London SE1 9SP, UK; roberto.sacco@manchester.ac.uk; 8 Research Center for Dental Sciences (CICO), Dental School, Universidad de La Frontera, Temuco 4811230, Chile; 9 Division of Oral, Facial and Maxillofacial Surgery, Dental School, Universidad de La Frontera, Temuco 4811230, Chile

**Keywords:** TMJ prostheses, condylar resorption, TMJ osteoarthritis, TMJ osteoarthrosis, TMJ arthritis, condylar degeneration

## Abstract

**Objectives**: This study aims to describe and analyze the indications and clinical results of total TMJ replacement in participants with degenerative and/or inflammatory joint diseases, defining patient and intervention conditions. **Methods**: A systematic review was conducted according to the Cochrane Handbook for Systematic Reviews of Intervention and reported according to the PRISMA Items update. The search strategy was from 1997 to July 2024 in Pubmed, Embase, Scopus, and Web of Science. A search for gray literature was conducted in the databases Google Scholar and Open Access Theses and Dissertations (OATD), and there were no limitations on the language or study design. We incorporated studies involving human patients over 15 years of age with degenerative and/or inflammatory joint conditions who underwent joint prosthesis replacement, either concurrently or separately from orthognathic surgery, as an initial intervention or after prosthesis installation. Participants with a postoperative follow-up of 12 months or longer were included. A risk of bias analysis was performed for non-randomized studies using the ROBINS-I tool, and GRADE profiler (GRADEpro) software was used to assess the quality of evidence and synthesize the data. **Results**: All the selected studies performed postoperative follow-up with quantitative and qualitative parameters; 10 performed a follow-up of 2 to 5 years. The indication for joint prosthesis replacement due to system failure was only 4.07%. Concerning diagnoses, 579 presented degenerative and/or inflammatory joint diseases, with osteoarthritis being the most frequent, followed by osteoarthrosis, juvenile idiopathic arthritis, and rheumatoid arthritis. The maximum mouth opening of the participants with TMJ disease presented an average of 24.32 ± 5.8 mm with a range of 18 to 36.4 mm. Of the 579 participants, the studies mention that they presented a soft to liquid diet and pain associated with decreased mandibular functionality. **Conclusions**: A total of 76.18% of the participants presented a range of moderate to severe pain associated with a decrease in functionality and, after joint replacement, all participants mentioned a decrease in pain or absence of pain, a change in diet by incorporating solid foods, and an increase in opening with an average of 40.74 ± 3.1 mm. Total joint replacement shows favorable long-term results. It is not possible to identify the best time to perform joint replacement surgery, considering the time since diagnosis of the disease, the time since the start of non-surgical treatment, or the number of previous surgeries.

## 1. Introduction

Temporomandibular joint (TMJ) disorders include a degenerative musculoskeletal disorder associated with morphological and functional deformities showing changes in the biological response to normal requirements. Usually, a lack in the balance of the joint structure, function, and occlusal relationships is observed, generating anomalies in the position and structure of the intra-articular disc and dysfunction of the related musculature [1]. A disc in a degenerative stage may present alterations in its fibrous structure, with possible thickening or gaps in the collagen matrix, calcifications, fibrosclerosis, or myxoid degeneration. Degenerative changes are more common in women and increase with aging and could be related to trauma, joint overload, or a secondary disease related to disc displacement [2].

The progression of joint disease may be due to degenerative joint involvement by inflammatory diseases such as synovitis capsulitis, polyarthritis (rheumatoid arthritis, psoriatic arthritis, ankylosing spondylitis, etc.), non-inflammatory diseases such as osteoarthritis or osteoarthrosis [3,4], or congenital developmental disorders such as juvenile idiopathic arthritis [5]. In degenerative joint diseases, the first joints affected are those with the highest mechanical load on the body weight and subjected to continuous stress and strain processes, such as the knee joint, the hip, and the spine. In the case of the TMJ, the alterations appear after the disc is displaced and there is contact between the condyle and the articular fossa, where the symptomatology is associated with the severity of the disease. Generally, diagnoses are made based on tests such as magnetic resonance and computed tomography (CT) or cone beam computed tomography (CBCT), which demonstrate the morphological alteration of the joint [6,7].

TMD and the associated pain are frequently encountered in clinical practice, negatively impacting the patient’s quality of life. However, various techniques are used to reduce pain; there is still controversy in selecting the most effective treatment, including surgical treatment of the TMJ [8].

A joint prosthesis consists of an implant that is surgically installed in the jaw and the cranial base to replace the TMJ and is considered as a radical surgical treatment when patients present symptoms of pain, difficulties with eating and jaw function, decreased mouth opening, and severe alterations in facial morphology [9]. Once installed, TMJ prostheses have a low failure rate with an incidence of 0.52% [10] due to their high wear resistance and good long-term biomechanical stability [11]. In severe TMJ diseases, a joint prosthesis will improve the patient’s quality of life in relation to pain and functional and social aspects [12].

This study aims to describe and analyze the clinical performance of total TMJ replacement in participants with degenerative and/or inflammatory joint diseases, defining patient and intervention conditions.

## 2. Materials and Methods

A systematic review was conducted per the Cochrane Handbook for Systematic Reviews of Interventions was reported following the updated Reference Items for Publication of Systematic Reviews (PRISMA) [13] to answer the following research question: What are the indications and clinical outcomes of total TMJ replacement in participants with degenerative and/or inflammatory joint disease? P: participants with degenerative and/or inflammatory joint disease of the TMJ; I: treatment by total TMJ replacement with prostheses; C: indications and use of TMJ prostheses; O: clinical outcomes after 12 months from the installation of the joint prostheses.

The search strategy covered PubMed, Embase, Scopus and Web of Science from 1997 to July 2024. A search for gray literature was conducted in the databases Google Scholar and Open Access Theses and Dissertations (OATD) and there were no limitations on the language or study design. Studies published from 1997 years onward were selected because the American Dental Federation published a final regulation in the Federal Register requiring manufacturers of TMJ implants to submit premarket approvals with data showing the safety and efficacy of the implants. We registered our protocol on PROSPERO, and the registration ID is as follows: 602516.

MeSH terms were used in the registered terms and the boolean terms AND/OR were used as follows: (Temporomandibular joint replace OR Temporomandibular joint prostheses OR Artificial temporomandibular joint) AND (condylar resorption OR TMJ osteoarthritis OR Temporomandibular Joint Osteoarthritis OR TMJ Osteoarthrosis OR Temporomandibular Joint Osteoarthrosis OR TMJ-OA OR TMJ arthritis OR Temporomandibular Joint arthritis OR condylar degeneration).

Data selection was carried out by two independent researchers calibrated using a Kappa index (0.617) in two weeks (V.R. and S.O.). After applying the search terms, duplicates were eliminated using Mendeley 2.90.0 software (Reference Management, Elsevier, London, UK). Articles were initially selected by evaluating the title and abstract according to the inclusion and exclusion criteria. Papers that appeared to meet the criteria were reviewed in full text by the same reviewers (V.R. and S.O.). In case of discrepancy, a consensus was reached by discussion with a third reviewer (R.S.).

All data were documented in an organized Excel spreadsheet. Authors were contacted for missing data, allowing a six-week response period. Data that were not available or missed were identified as “not described” (ND) in the tables.

**Figure 1:**
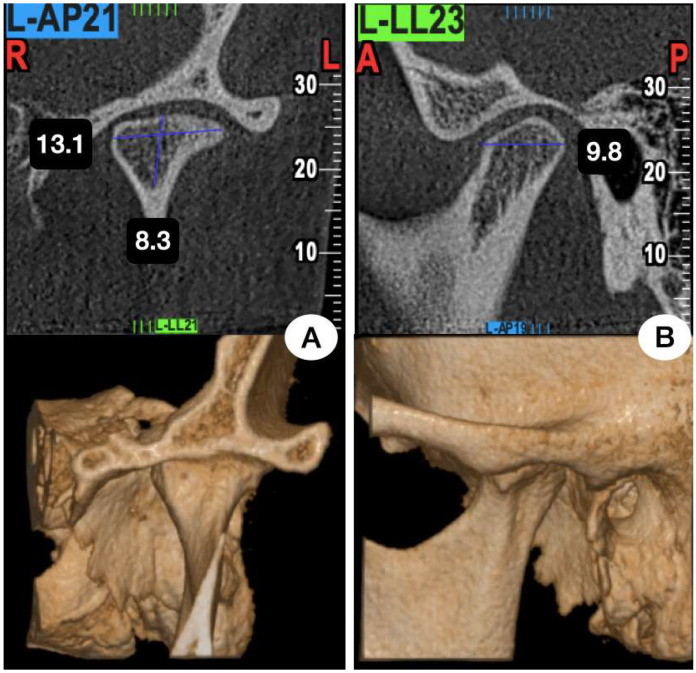
CBCT frontal (**A**) and sagittal (**B**) sections of TMJ with degenerative process and morphological change of the condyle.

The studies involved human patients over 15 years of age with degenerative and/or inflammatory joint problems (Figure 1) who received joint replacement prostheses, either simultaneously or not with orthognathic surgery, either as initial treatment or as treatment after prosthesis installation; participants with a follow-up of 12 months or more in the postoperative stage were included. If the selected study presented other joint pathologies in the approach, only articles with 50% of the sample comprising degenerative and/or inflammatory joint diseases were selected. Studies with samples of fewer than 10 patients or that used animals or cadavers and were not in full text were excluded.

Independently, a risk of bias analysis was performed for non-randomized studies using the ROBINS-I tool [14]. The risk of bias was subdivided into 7 categories, (1) confounding, (2) study participant selection, (3) exposure measurement, (4) post-exposure interventions, (5) missing data, (6) outcome measurement, and (7) outcome reporting, and each category was scored as having a low, moderate, or serious (critical) risk of bias or no information. Furthermore, GRADE profiler (GRADEpro, version 7.11.0) software was used to assess the quality of evidence and synthesize the data, as advised by the Cochrane Collaboration and the GRADE Working Group [15]. High: we are certain that the actual impact is near the estimated effect. Moderate: we have a moderate level of confidence in the impact estimate. Low: we expect the actual impact to approximate the estimated effect, but there is a risk of significant divergence. Very low: We have limited confidence in the impact estimate. The actual impact may significantly diverge from the estimated effect.

## 3. Results

**Figure 2:**
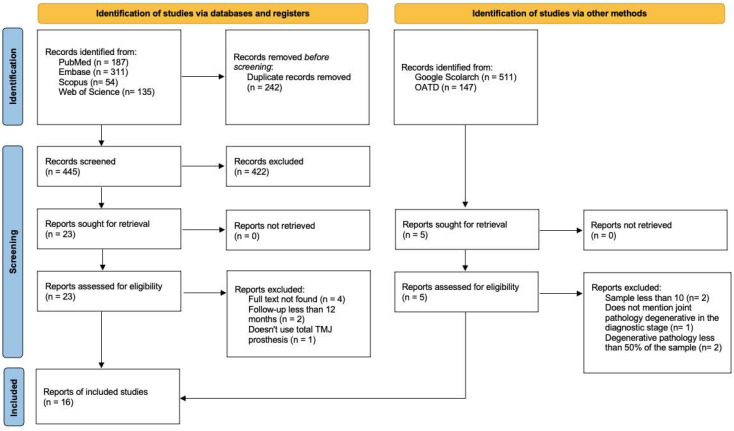
Flow chart of the systematic review.

The systematic review identified 687 articles. After eliminating 242 duplicates, 445 articles were chosen for title and abstract review, and applying the inclusion and exclusion criteria, 23 papers were identified for comprehensive study (Figure 2). Of the 23 articles, 7 studies were excluded for not meeting the inclusion criteria; 4 articles [16,17,18,19] were not in full text, 2 articles [20] presented a sample less than 12 months, and 1 article [21] did not use total TMJ prostheses as a definitive treatment. The search for gray literature yielded 665 documents; 650 were excluded through title and abstract analysis. Of the five selected articles, all were excluded because they did not meet our inclusion criteria. A total of 16 studies [22,23,24,25,26,27,28,29,30,31,32,33,34,35,36,37] were analyzed in this article.

The selected articles included a total of 760 participants. The age range of the included studies ranged from 15 to 75 years, with an average of 39.7 ± 9.16 years. A total of 78 (10.26%) participants were men and 682 (89.73%) were women. All the selected studies had follow-ups ranging from 12 to 120 months. Concerning the country where the study was conducted, there is a higher prevalence of studies conducted in the United States (50%), followed by the United Kingdom (31.24%), Australia, Canada, and China. Eight studies were prospective in design, and eight were retrospective. Twelve studies presented a quasi-experimental design, two studies (Table 1) [24,33] mentioned a cohort design, while two studies [34,36] had a non-randomized clinical trial design, but none had blinding of the investigators. TMJ Concepts prostheses were the most used, followed by Zimmer Biomet and Christensen. A total of 1231 TMJ prostheses were fitted; 874 were customized, and 357 were stock prostheses. In addition, 828 were bilateral prostheses, and 308 were unilateral, with a higher prevalence of installation on the left side.

All selected studies performed postoperative follow-ups with quantitative and qualitative parameters; 10 studies performed a follow-up after 2 to 5 years [22,23,24,26,27,29,30,32,33,36] and 4 studies followed up between 5 and 10 years [28,34,35,37]. All studies report changes between the pre-surgical stage and the post-surgical stage. However, no significant changes were observed in relation to the maximum incisal opening, diet consistency, and pain in evaluations after 12 months or 5 years among the quantitative, qualitative, and imaging parameters. Concerning the functioning of the prostheses between 5 and 10 years, the studies indicate no complications in mandibular functionality and no failures in the joint prostheses (Table 2).

Table 3 presents characteristics at the diagnostic stage, where the indication for joint prosthesis replacement due to system failure was only present in 31 participants (4.07%) out of 760. In relation to the diagnosis, of the 760 participants, 579 (76.18%) presented degenerative and/or inflammatory diseases in the joint, with osteoarthritis being the most frequent, followed by osteoarthrosis, juvenile idiopathic arthritis, and rheumatoid arthritis. The maximum mouth opening of the participants with TMJ disease presented an average of 24.32 ± 5.8 mm with a range of 18 mm to 36.4 [28,34]. Of the 579 participants, the studies mention that they presented a diet in soft to liquid consistencies; moreover, all presented pain, 574 participants presented a range of moderate to severe pain associated with decreased mandibular functionality, 123 presented moderate pain, and 21 participants presented mild to moderate pain. In contrast, in one study [25] with a sample of 42 participants, no clinical features were present at the diagnostic stage.

The diagnostic methods that presented the highest frequency were computed tomography and magnetic resonance imaging. Studies by Sidebottom and Gruber [25], Gruber et al. [27], O’Connor et al. [29] and Rajkumar and Sidebottom [35], in addition to performing imaging analyses, also incorporated histopathologic analysis following joint removal to corroborate the diagnosis.

With treatments prior to TMJ prosthesis replacement, nine studies [22,23,25,27,29,32,34,35] with a sample of 619 participants describe the interventions before prostheses installation, where 327 (52.82%) underwent surgical interventions such as arthroscopies, discectomies, and steroid infiltration in conjunction with condylar repositioning procedures. Meanwhile, the study by Sarlabous et al. [33] describe conservative treatment with drugs alone in 39 participants prior to joint replacement surgery. In 210 (33.92%), joint prostheses installations were performed with no previous pharmacological or surgical treatment.

**Figure 3:**
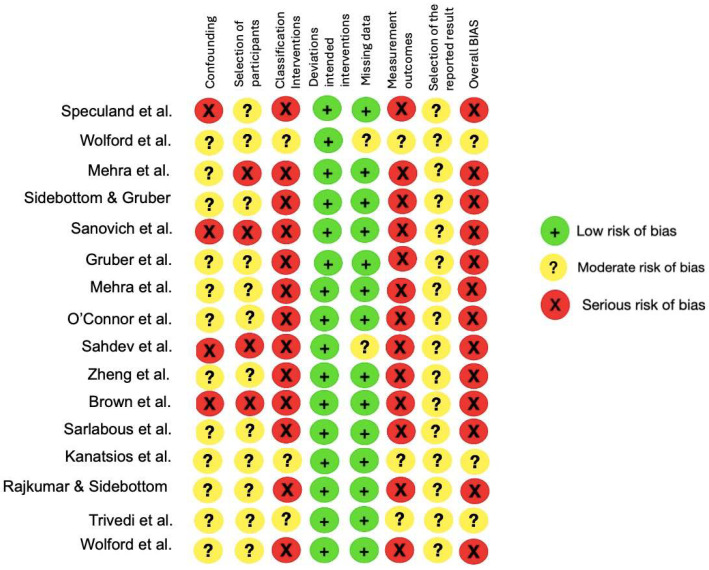
Summary of risk of bias of the included studies (green: strong; yellow moderate; red: weak) [22,23,24,25,26,27,28,29,30,31,32,33,34,35].

Regarding the risk of bias, the 16 selected articles were evaluated with the ROBINS-I tool (Figure 3). In terms of the confounding bias item, 12 studies presented moderate bias due to the lack of blinding by the evaluators, surgeons, and processing of results. In comparison, four studies demonstrated a severe risk of bias because the follow-up of the controls was in a different follow-up time. In terms of selection bias, four studies had a serious risk because the initial and final treatment samples were different due to dropout, especially the months of the second or third control, which had different samples. In relation to the classification of the intervention, three studies presented moderate risk because they had intervention groups for both joint pathologies and testing of different prostheses, while 12 studies had severe risk because they were quasi-experimental. All the studies had a low risk of deviation bias in the interventions since they all evaluated the response to treatment and described favorable and unfavorable outcomes. Only two studies excluded participants due to a lack of data during postoperative check-ups. Data collection and processing methods were low risk in all studies because the inclusion and exclusion criteria were specific.

On the other hand, according to the quality of the evidence using the five GRADE criteria, all selected studies were categorized as low-quality due to a significant risk of bias due to at least one domain being highlighted as high-risk in each study. Consequently, the assessments of the findings’ impact are ambiguous, and the published results need careful evaluation (Table 4).

**Figure 4:**
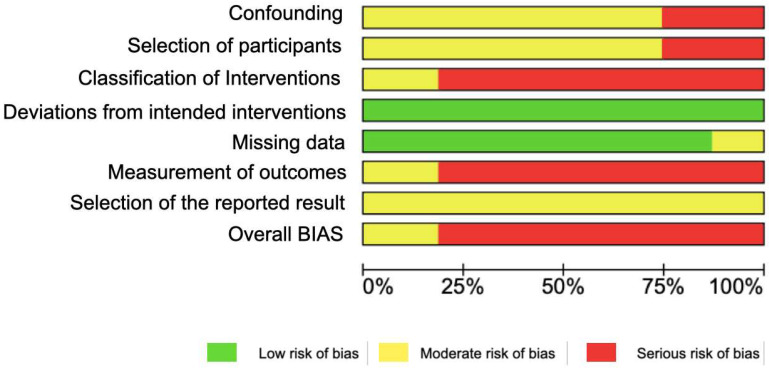
Overall percentage of risk of bias of the included studies (green: strong; yellow: moderate; red: weak).

In the overall ranking (Figure 4), three studies were at moderate risk due to weak study designs, mainly blinding, while two were at a high risk of bias.

**Assessment of heterogeneity:** The Cochran Q test was used to assess whether the studies included in the systematic review show variability that exceeds random variability, indicating that the studies are not homogeneous. If the null hypothesis is rejected (null hypothesis (H0): there is no heterogeneity between studies (all studies have the same effect)), then the alternative hypothesis (H1) is accepted: there are significant differences between studies (there is heterogeneity). In relation to our data, it was observed that there is significant heterogeneity between the studies included in the review (*p* = 0.001). This means that the differences in the results of the studies are not due to chance alone, but that there is some systematic factor or factors that are causing variability in the observed effects. This result does not imply that the studies are incorrect or invalid, but that the studies are different, generating bias when comparing results between studies. Meta-analysis is a powerful tool in scientific research to combine and analyze the results of previous studies, seeking to obtain more robust and generalizable conclusions. However, in our study, we observed a lack of quality in the analyzed studies, non-compatibility in measurements and variables, a lack of available data and information, and study designs that are not suitable for a meta-analysis due to heterogeneity in the interventions, which leads to a lack of interpretation and application of the results. Some studies do not describe statistical results in relation to their measures of dispersion or central tendency, and the quality of the data or the reporting of results may be insufficient or unreliable for synthesizing them quantitatively, since qualitative measurements were common and quantitative analyses showed differences in terms of measurements and time to assess treatment progress.

## 4. Discussion

The frequency of temporomandibular joint diseases spans a broad age spectrum; 20-year-old individuals may exhibit asymptomatic cases, but those over 40 often experience mild and variable pain, typically exacerbated by chewing or other mandibular functions [31]. In aging, the functional demands on the TMJ may exceed the joint’s capacity for repair and remodeling, resulting in a degenerative joint disorder with a higher prevalence in women than men [38,39]. In our study, we observed a mean of 39.7 ± 9.16, where women presented a higher prevalence of joint disease than men. These data agree with the study by Segal et al. [40], where women represent 60% of the sample with joint diseases, with a greater difference after 40 years. Furthermore, they present greater limitations in physical function than men regardless of body mass index, severity, injury history, or the amount of weekly exercise.

Joint replacement in patients with joint disease can be complicated by altered anatomy and infiltration of inflamed tissue into the joints [41]. In the same way, systemic disease, immunosuppression, and functional constraints can influence both short- and long-term anatomical conditions, as localized and periarticular bone loss occurs, produced by the suppression of osteoclasts and osteoblasts [42]. Patients with rheumatoid arthritis, psoriatic arthritis, or ankylosing spondylitis are likely to experience accelerated progression in the synovial joints due to proinflammatory cytokines, nitric oxide, matrix-degrading enzymes, and biomechanical stress. This will necessitate alloplastic replacement treatments via joint prostheses as inflammation persists unabated [43,44]. Based on the data obtained in our study, we noted that among the characteristics to indicate a joint replacement, diseases such as osteoarthritis, followed by osteoarthrosis, juvenile idiopathic arthritis, and rheumatoid arthritis, are the most prevalent. In these patients, there was an opening range of 24.32 ± 5.8 mm in the preoperative stage, and it was related to a frequency of food consumption in soft and liquid consistencies, together with decreased mandibular functionality due to moderate to severe pain.

Owing to the controversy of diagnostic criteria, various procedures are used for TMJ pain or dysfunction, with minimally invasive techniques constituting approximately 90% of all treatments, while only 10% of cases persist with painful mandibular dysfunction necessitating surgical intervention, such as joint replacement [45]. Minor joint surgeries can reduce pain by eliminating inflammatory cells from the joint space and increasing mandibular mobility by removing intra-articular adhesions [46]. This type of treatment shows positive results in early-stage joint problems with a 6-year follow-up, whereas, in advanced stages, it only generates temporary relief [47]. Of the selected articles, in 43.02% of the sample, joint interventions were performed prior to total TMJ replacement, where eight studies [22,23,25,26,27,30,34,35] do not describe exactly what type of intervention was performed, while the studies by O’Connor et al. [29] and Brown et al. [32] report that arthrocentesis and arthroscopy were performed as previous treatments. No study mentions the frequency and time of joint replacement treatments performed prior to joint replacement. Still, they make it evident that for reasons of pain and limited functionality, it was necessary to install the TMJ prostheses.

Alterations in the joint and mandibular condyle significantly influence the size, shape, and function of the mandible; if left untreated, acquired TMJ abnormalities can lead to facial deformities over time due to the remodeling process of the TMJ. This results in reduced mandibular displacement that affects the posterior vertical position of the mandible [48,49]. Hsieh et al. [50] performed an analysis of facial morphology in participants with juvenile idiopathic arthritis with moderate to severe TMJ involvement vs. participants with juvenile idiopathic arthritis without TMJ involvement; they observed that patients with joint involvement had a facial deformity with reduced posterior mandibular height, chin retrusion, and prominent upper buccal area. Given these characteristics, it may be necessary to incorporate complementary surgical procedures to joint replacement, where orthognathic surgeries could correct dentofacial deformities and asymmetries and improve neuromuscular and occlusal coordination [51]. Using a systematic review with meta-analysis, Kim et al. [52] demonstrated positive results regarding skeletal improvements, long-term stability, psychological well-being, and quality of life using orthognathic surgery.

Treatments employing articular prostheses are protocolized procedures, defined, stable, and predictable, which allow for positive results in pain reduction and better movement and mandibular function [53]. Due to the presence of facial asymmetries resulting from joint alterations, incorporating complex midfacial and mandibular surgeries performed simultaneously with total TMJ replacement makes it possible to restore mandibular function and facial morphology; however, its success depends mainly on adequate planning and execution [54]. In our review, nine articles performed only articular prosthetic reconstructive surgeries, while seven studies performed orthognathic surgeries in conjunction with articular prostheses, equivalent to 24.47% of the total sample. In addition, of the 186 participants who underwent orthognathic surgery, 91.65% of the sample had bilateral prostheses.

Joint prosthesis is considered a viable treatment compared to autogenous grafts for TMJ reconstruction [55]. Lima et al. [56] evaluated the survival of joint replacements through a systematic review with meta-analysis, observing that post-surgical complications occur within the first 6 months, with infection being the main cause, while a follow-up of 12 months or more observed a 97% survival rate. Studies by Wolford et al. [57] and Mercuri et al. [58] show that joint prostheses over a long-term use of 10 to 14 years are safe, effective, and reliable, where all participants mentioned improvements in the quality of life in the absence of clinical situations with the need for changes to the joint prostheses. The main disadvantage of these studies is the low long-term follow-up of patients. Wolford et al. [57] only managed to monitor 55 participants through questionnaires and measurement of maximum incisal opening, while Mercuri et al. [58] conducted surveys via e-mail.

All the studies included in this review presented follow-ups after 12 months of treatment through surveys or clinical and radiographic analyses, showing changes in the maximum incisal opening, a change from liquid or soft to solid consistencies in diet and the absence of or highly reduced pain. In follow-ups longer than 12 months, we observed that the sample decreases, as in the study by Speculand et al. [22], where at 120 months, only 3 patients had a check-up, while O’Connor et al. [29] performed a check-up on 19 patients at 105 months and Trivedi et al. [36] evaluated 12 participants over 132 months. Only two studies [34,35] conducted follow-ups extending to 10 years using surveys. Kanatsios et al. [34] monitored 28 participants, and Rajkumar and Sidebottom [35] monitored 43 participants. Studies with follow-ups of up to 5 years mention that there is a decrease in the maximum incisal opening within a range of 2 to 5 mm but that this does not represent clinical changes, while in studies that carry out follow-ups of more than 5 years, they mention that they observe changes between 0.5 and 1 mm. While all the studies show clinical improvements and quality of life after joint replacement, no study mentions having made joint changes or the need to make them.

Several studies [56,57,58] demonstrate the efficacy, safety, and stability of joint prostheses in a range of 5 to 20 years, mentioning that although there is a decrease in the maximum incisal opening, these changes are not clinically significant, in addition to obtaining better conditions in functionality and pain reduction. Despite the favorable results obtained in relation to joint replacement, in our studies, we observed a heterogeneity in the diagnosis for the indication of joint replacement. In addition, most of the articles lacked a control group, being studies with quasi-experimental designs with no blinding of the researchers.

## 5. Conclusions

Patients with degenerative joint issues associated with inflammatory or autoimmune conditions, accompanied by pain and mandibular dysfunctions, benefit from total joint replacement with prosthetic joints, yielding favorable long-term outcomes. Overall, 76.18% of participants presented a range of moderate to severe pain associated with a decrease in functionality, with a maximum incisal opening range of 24.32 ± 5.8 mm. After joint replacement, all participants mentioned a decrease in pain or the absence of pain, a change in diet by incorporating solid foods, and an increase in opening with an average of 40.74 ± 3.1 mm.

It is not possible to identify the best time to perform joint replacement surgery, due to the time elapsed since the diagnosis of the disease, the time elapsed since the start of non-surgical treatment, or the number of previous surgeries. A joint prosthesis facilitates morphological and functional reconstruction in instances of significant functional impairment and can be integrated with orthognathic surgery to enhance the overall treatment conditions.

## Figures and Tables

**Table 1:** Description of the 16 studies included in the systematic review regarding objectives and methodology.

Author	Objective	Country	Design of the Studies
Speculand et al. [22]	The objective was to present the clinical experience of the total TMJ prosthesis system at two centers in the United Kingdom over a 10-year follow-up period.	United Kingdom	Prospective
Wolford et al. [23]	To evaluate outcomes over 5 to 8 years in 42 patients who underwent temporomandibular joint reconstruction using TMJ Concepts’ customized total joint prostheses.	United States	Prospective
Mehra et al. [24]	To evaluate the results of the single-stage reconstruction of rheumatoid arthritis patients with pathologic features of the TMJ and an associated dentofacial deformity.	United States	Prospective
Sidebottom & Gruber, [25]	To describe the results and complications after total TMJ replacement with the TMJ Concepts system.	United Kingdom	Prospective
Sanovich et al. [26]	To report the outcomes for patients operated on with the Biomet micro-fixation TMJ replacement system at the University of Florida.	United States	Retrospective cohort
Gruber et al. [27]	To evaluate the medium-term benefits, efficacy, and safety of the TMJ Concepts joint replacement system in the UK.	United Kingdom	Prospective
Mehra et al. [28]	The objective of this study was to report clinical outcomes after TMJ prosthetic replacements for managing idiopathic condylar resorption.	United States	Retrospective
O’Connor et al. [29]	They compared the outcomes of subjects who had inflammatory arthritis with those who had non-inflammatory joint degeneration after total TMJ replacement with TMJ Concepts System prostheses.	United Kingdom	Prospective
Sahdev et al. [30]	This study aimed to evaluate changes in pain, mandibular range of motion, and postoperative complications and comorbidities in subjects undergoing total joint replacement at Massachusetts General Hospital.	United States	Retrospective
Zheng et al. [31]	This study aimed to evaluate the safety and efficacy of the new custom-designed, 3D-printed additive-manufactured TMJ prostheses in clinical application.	China	Prospective
Brown et al. [32]	The objective was to evaluate the efficacy of total joint replacement with alloplastic total joint prostheses in patients with juvenile idiopathic arthritis.	United States	Retrospective
Sarlabous et al. [33]	To demonstrate the results of total TMJ replacement with alloplastic devices in patients with inflammatory arthritis.	Canada	Retrospective
Kanatsios et al. [34]	This study aimed to compare the clinical outcomes of Zimmer-Biomet TMJ prostheses with the customized OMX TMJ prostheses in patients with osteoarthritis of the TMJ.	Australia	Retrospective
Rajkumar & Sidebottom, [35]	The objective was to evaluate the long-term benefits of the TMJ Concepts joint replacement system in the United Kingdom.	United Kingdom	Prospective
Trivedi et al. [36]	The objective was to evaluate the surgical outcomes of patients with idiopathic juvenile arthritis of the TMJ after reconstruction with customized total joint replacement and orthognathic surgery.	United States	Retrospective cohort
Wolford et al. [37]	To determine surgical changes and long-term stability outcomes in subjects with diagnoses of juvenile idiopathic arthritis after TMJ reconstruction with customized total prostheses and orthognathic surgery.	United States	Retrospective cohort

**Table 2:** Characteristics of the 16 articles regarding clinical characteristics.

Author	N	Sex (M/F)	Age (Years)	Complementary Surgery	No. of Prostheses Installed	Prostheses Manufacture	Follow-Up (Months)
Speculand et al. [22]	62	9–53	Mean age 44 years	ND	86 TMJ prostheses (27 Vitek VK II prostheses and 59 Christensen prostheses). 48 bilateral; 16 left unilateral; 22 right unilateral.	The Vitek VK II system and Christensen prostheses	28 participants for 12 months, 17 for 24 months, 9 for 48 months, 3 for 60 months, 2 for 10 months, and 3 for 120 months.
Wolford et al. [23]	38	1–37	Mean age 36 years (15 to 64 years)	ND	69 TMJ prostheses	TMJ Concepts Custom Prosthetics	Average 73.5 months (60 to 96 months)
Mehra et al. [24]	15	3–12	Mean age 27.4 (15 to 61 years)	10 participants underwent orthognathic surgery and 5 participants underwent only mandibular osteotomy	30 bilateral TMJ prostheses	TMJ Concepts Custom Prosthetics	34.3 months
Sidebottom & Gruber, [25]	74	9–65	Mean age 47 (19 to 72 years old)	Two orthognathic surgeries were performed to level the occlusal plane and correct the skeletal Class II rotation.	103 prostheses: 29 bilateral; 18 right unilateral; 27 left unilateral	TMJ Concepts Custom Prosthetics	12 months
Sanovich et al. [26]	36	0–36	Mean age 49.4 ± 11.9	ND	62 prostheses: 52 bilateral; 6 left unilateral; 4 right unilateral	Biomet stock prostheses	30 months
Gruber et al. [27]	58	6–52	Mean age 47 (19 to 72 years)	ND	84 prostheses: 52 bilateral; 20 left unilateral; 12 right unilateral	TMJ Concepts Custom Prosthetics	At 36 months, 58 participants were followed up; at 60 months, 26 participants were followed up.
Mehra et al. [28]	21	0–21	Mean age 25.6 (22 to 32 years)	16 participants underwent orthognathic surgery for the presence of skeletal class II facial deformity and open bite.	42 bilateral prostheses	TMJ Concepts Custom Prosthetics	On average, 74.4 months (60 to 144 months)
O’Connor et al. [29]	22	0–22	Mean age 40 (16 to 71 years)	ND	39 prostheses: 34 bilateral; 3 right unilateral; 2 left unilateral.	TMJ Concepts prostheses	22 participants were evaluated at 12 months, and 14 were evaluated from 19 to 105 months.
Sahdev et al. [30]	95	10–85	Mean age 44.3 ± 11.82 (18 to 75 years)	ND	175 prostheses: 134 bilateral; 41 unilateral	TMJ Concepts Custom Prosthetics	52.8 ± 3.38 months
Zheng et al. [31]	12	5–7	Mean age 47.8 (35 to 66 years)	ND	12 prostheses: 7 right unilateral; 5 left unilateral	Customized and manufactured prostheses	12 months
Brown et al. [32]	20	1–19	Mean age 18 years (16 to 23 years old)	11 participants underwent orthognathic surgery	40 prostheses: 38 bilateral; 2 unilateral	TMJ Concepts Custom Prosthetics	Average 30.9 months (12 to 92 months)
Sarlabous et al. [33]	39	7–32	Mean age 36 (18 to 61 years)	Le Fort I osteotomy and genioplasty (only described when necessary, without indicating the number of participants who underwent the procedure)	74 prostheses: 70 bilateral; 4 unilateral	5 participants were with TMJ Concepts prostheses; 1 subject with Christensen Prostheses; 20 with customized Zimmer-Biomet prostheses; 13 participants with stock prostheses.	33 participants to 12 months. On average, 45.9 follow-up
Kanatsios et al. [34]	117	7/110	Mean age 53.4 ± 12.7 years	ND	139 prostheses: 44 bilateral; 95 unilateral	54 participants with Zimmer Biomet stock prostheses; 63 participants with custom OMX prostheses.	98 participants to 12 months; 52 were evaluated at 5 years and 28 at 10 years.
Rajkumar & Sidebottom, [35]	43	4–39	Mean age 45 years (22 to 70 years)	ND	62 prostheses: 38 bilateral; 17 left; 7 right	TMJ Concepts Custom Prosthetics	Follow-up of 43 participants at 3 years; follow-up of 26 participants at 5 years; follow-up of 43 participants at 10 years.
Trivedi et al. [36]	66	9–57	Mean age between 17.5 and 35.5 years	66 orthognathic surgery with mandibular advancement	132 bilateral prostheses: 80 in participants with juvenile idiopathic arthritis and 52 in participants with degenerative disease not associated with juvenile idiopathic arthritis.	TMJ Concepts Custom Prosthetics	Follow-up for the juvenile idiopathic arthritis group was 26.5 months (range 12 to 236 months), and for the degenerative disease group, 24 months (range 12 to 143 months).
Wolford et al. [37]	42	7–35	Mean age 17.5 ± 9.8	42 Orthognathic surgery with mandibular advancement; 36 genioplasty	82 bilateral prostheses.	TMJ Concepts Custom Prosthetics	35.3 months (range 12 to 114 months)

Obs: ND: Not Describe.

**Table 3:** Clinical and imaging characteristics of the 16 studies included in the pre-surgical stage, as well as the presence of prior interventions.

Author	Maximum Incisal Opening in Diagnostic Stage	Pain, Difficulty with Eating or Mandibular Movement at Diagnostic Stage	Complementary Diagnostic Methods	Diagnosis of the Sample	Treatments Prior to TMJ Prostheses
Speculand et al. [22]	ND	60 participants had moderate to severe pain (5–10); 48 participants could only eat liquid and soft foods.	Magnetic resonance imaging and computed tomography analysis	33 with arthropathy or osteoarthritis; 10 with rheumatoid arthritis; 9 with ankylosis; 6 with severe joint dysfunction; 2 with replacement of joint prostheses; 1 with psoriatic arthritis	25 participants had undergone previous surgery without favorable outcomes.
Wolford et al. [23]	27.5 mm	On average, they presented moderate to severe pain (7.7); difficulty in mandibular movement was reduced (7.1).	Computed tomography analysis	The type of degenerative TMJ disease is not described, but all had previous TMJ surgery.	All had previous TMJ surgery.
Mehra et al. [24]	34.6 mm	On average, they presented moderate pain (6.7), a soft diet, and decreased mandibular function (7.8).	Magnetic resonance imaging and computed tomography analysis	15 with rheumatoid arthritis	No previous TMJ treatments were performed
Sidebottom & Gruber, [25]	22 mm	On average they presented moderate to severe pain (7.2); soft to liquid diet.	Analysis by computed tomography and confirmed by histopathological examination after joint replacement.	27 with degenerative disease; 13 with multiple operations; 10 with rheumatoid arthritis; 11 associated with condylar lesions; 12 with ankylosis; 10 previous TMJ prostheses	13 participants had previous surgical procedures.
Sanovich et al. [26]	26.1 mm	On average, they had moderate to severe pain (7.9) and a soft to liquid diet.	ND	15 with degenerative disease; 4 with rheumatoid arthritis; 7 with ankylosis; 6 with TMJ prostheses failure; 3 due to trauma; 1 due to pathology.	6 participants had TMJ prostheses that had to be replaced
Gruber et al. [27]	21 mm	On average, they presented moderate to severe pain (7.4); soft to liquid diet	Analysis by computed tomography and confirmed by histopathological examination after joint replacement.	15 degenerative disease; 11 arthritis; 11 post-trauma; 8 TMJ prostheses failure; 7 ankylosis; 6 TMJ surgeries	The average number of previous surgeries per patient was 2.4.
Mehra et al. [28]	18.6 mm	On average, they had mild to moderate pain (3.2); soft to liquid diet	Analysis by panoramic radiography and cephalometry	21 participants with idiopathic condylar resorption.	ND
O’Connor et al. [29]	23 mm	On average, they presented moderate pain (5.5 to 6.2); a soft diet	Analysis by computed tomography and confirmed by histopathological examination after joint replacement.	17 rheumatoid arthritis; 9 psoriatic arthritis and/or ankylosing spondylitis	The previous treatments were discectomy and TMJ arthroscopy with and without steroids, and only one patient underwent disc replacement and application with condylar wear.
Sahdev et al. [30]	25.4 mm	72 participants presented moderate to severe pain (6.9).	ND	42 ankylosis; 22 inflammatory disease; 18 degenerative disease; 12 trauma; 1 deformity or congenital abnormality	Overall, patients had a mean of 4.7 ± 5.42 previous surgical procedures.
Zheng et al. [31]	26.42 mm	On average, they presented moderate to severe pain (7.1), a soft to liquid diet, and decreased mandibular function (6.0).	Computed tomography analysis	12 osteoarthrosis	ND
Brown et al. [32]	30.55 to 32.9 mm	On average, they presented moderate pain (5.7), a soft to liquid diet, and moderate mandibular function.	All were diagnosed by a pediatric rheumatologist.	20 participants with juvenile idiopathic arthritis.	Intra-articular steroid injections, arthrocentesis and arthroscopy were performed in 17 participants.
Sarlabous et al. [33]	22.1 mm	On average, they presented moderate to severe pain (6.8).	Analysis by computed tomography and consultation with a rheumatologist.	21 rheumatoid arthritis; 4 psoriatic arthritis; 5 ankylosing spondyloarthritis; 5 juvenile idiopathic arthritis; 3 lupus; 1 mixed connective tissue disease	Conservative treatments were carried out with drugs according to diagnosis (Arava, Enbrel, Plaquenil, and nonsteroidal anti-inflammatory drugs).
Kanatsios et al. [34]	31.5 mm	On average, they presented moderate to severe pain (6.2)	ND	117 participants with osteoarthritis	54 participants presented with minimally invasive TMJ surgery.
Rajkumar & Sidebottom, [35]	21 mm	On average, they presented moderate to severe pain (7.4); soft to liquid diet	Analysis by computed tomography and confirmed by histopathological examination after joint replacement.	43 participants: 13 degenerative disease; 7 post-trauma; 5 TMJ prosthesis replacement; 3 rheumatoid arthritis; 5 ankylosis; 5 TMJ surgeries; 4 psoriatic arthritis; 1 ankylosing spondyloarthritis	5 participants had multiple joint surgeries
Trivedi et al. [36]	35.2 to 36.4 mm	On average, they presented moderate pain (4–6), a soft to liquid diet, and moderate mandibular function.	Magnetic resonance imaging and computed tomography analysis	40 participants with juvenile idiopathic arthritis; 16 with arthritis; 6 with condylar resorption; 3 with rheumatoid arthritis; 1 with psoriatic arthritis.	ND
Wolford et al. [37]	ND	ND	Magnetic resonance and lateral radiography analysis	42 participants with juvenile idiopathic arthritis.	ND

Obs: ND: Not Describe.

**Table 4:** Results of the analysis of the 16 studies using the GRADE tool to assess evidence levels.

Study	Quality Assessment					Grade of Evidence
Authors	Risk of Bias	Inconsistency	Indirectness	Imprecision	Other Considerations	Quality
Speculand et al. [22]	Serious	Not serious	Not serious	Serious	None	⊕⊕⊖⊖ Low
Wolford et al. [23]	Serious	Not serious	Not serious	Serious	None	⊕⊕⊖⊖ Low
Mehra et al. [24]	Serious	Not serious	Not serious	Not serious	None	⊕⊕⊖⊖ Low
Sidebottom & Gruber, [25]	Serious	Not serious	Not serious	Serious	None	⊕⊕⊖⊖ Low
Sanovich et al. [26]	Serious	Not serious	Not serious	Serious	None	⊕⊕⊖⊖ Low
Gruber et al. [27]	Serious	Not serious	Not serious	Not serious	None	⊕⊕⊖⊖ Low
Mehra et al. [28]	Serious	Not serious	Not serious	Serious	None	⊕⊕⊖⊖ Low
O’Connor et al. [29]	Serious	Not serious	Not serious	Serious	None	⊕⊕⊖⊖ Low
Sahdev et al. [30]	Serious	Not serious	Not serious	Serious	None	⊕⊕⊖⊖ Low
Zheng et al. [31]	Serious	Not serious	Not serious	Serious	None	⊕⊕⊖⊖ Low
Brown et al. [32]	Serious	Not serious	Not serious	Not serious	None	⊕⊕⊖⊖ Low
Sarlabous et al. [33]	Serious	Not serious	Not serious	Not serious	None	⊕⊕⊖⊖ Low
Kanatsios et al. [34]	Serious	Not serious	Not serious	Serious	None	⊕⊕⊖⊖ Low
Rajkumar & Sidebottom, [35]	Serious	Not serious	Not serious	Not serious	None	⊕⊕⊖⊖ Low
Trivedi et al. [36]	Serious	Not serious	Not serious	Serious	None	⊕⊕⊖⊖ Low
Wolford et al. [37]	Serious	Not serious	Not serious	Serious	None	⊕⊕⊖⊖ Low

Obs: ⊕: Strong recommendation; ⊖: Weak or conditional recommendation.

## Data Availability

The data are available upon request from the corresponding author.
